# Trigeminal Neuropathic Pain With Complicated Pathophysiology Caused by a Low-Level Impacted Mandibular Third Molar: A Case Report

**DOI:** 10.7759/cureus.84308

**Published:** 2025-05-17

**Authors:** Yoko Kudo, Seiji Asoda, Wataru Muraoka, Koichi Wajima, Taneaki Nakagawa

**Affiliations:** 1 Department of Dental Anesthesiology, Faculty of Dental Medicine and Graduate School of Dental Medicine, Hokkaido University, Sapporo, JPN; 2 Department of Dentistry and Oral Surgery, Keio University School of Medicine, Tokyo, JPN; 3 Department of Dentistry and Oral Surgery, Kawasaki Municipal Ida Hospital, Kawasaki, JPN

**Keywords:** amitriptyline, orofacial pain, pericoronitis, peripheral sensitization, pregabalin, trigeminal neuropathic pain

## Abstract

We report a case of neuropathic pain affecting the third branch of the trigeminal nerve caused by periodontitis associated with a low-level impacted mandibular third molar on the left side. The patient also experienced neuropathic pain in the second branch, which may have resulted from sensitization of the third branch. A 57-year-old woman presented with percussion and contact pain in the left maxillary second molar, along with burning pain extending from the left buccal region to the mandible. Pus discharge was noted from the periodontal pocket, and she was treated with anti-inflammatory medication and antibiotics. Although the pus discharge resolved, the pain persisted. The percussion and contact pain in the left maxillary second molar, as well as the burning pain from the left buccal area to the mandible, were diagnosed as neuropathic pain involving the left third branch of the trigeminal nerve, originating from inflammation of the left mandibular third molar. Persistent pain and allodynia in the second branch region were attributed to sensitization of the third branch. Pregabalin was added to her treatment, resulting in symptom relief. Imaging showed that the left mandibular third molar was low-level impacted and in close proximity to the inferior alveolar canal, which was identified as the source of the initial pain. The tooth was extracted under general anesthesia after pain symptoms improved. One month post-extraction, the patient experienced a flare-up of burning pain in the left maxillary second molar region and allodynia in the gingiva, as well as burning pain from the left buccal region to the mandible. Pregabalin was restarted but did not provide analgesic effects. Amitriptyline was then added, leading to pain relief. This case highlights the complex pathophysiology of neuropathic pain, which remains incompletely understood. Early intervention to relieve pain may be crucial to prevent chronic symptoms.

## Introduction

Traumatic nerve injury caused by tooth extraction or dental implants is a major cause of neuropathic pain in the orofacial region, with the incidence following third molar extraction reported at 0.38% [1] and that related to dental implants at 0.3% [2]. The International Association for the Study of Pain currently defines neuropathic pain as “pain caused by a lesion or disease of the somatosensory nervous system,” and its diagnostic criteria are widely used [3,4]. However, clinical findings play a crucial role in the diagnosis of “possible” neuropathic pain [5].

Confirming neuropathic symptoms requires assessment of additional sensory findings, such as pain symptoms that align with neuroanatomical patterns, a clinical history indicative of neuropathy, sensory abnormalities like allodynia, and imaging studies. Importantly, treatment should be initiated as early as possible, even at the stage of suspected diagnosis, regardless of whether other findings are present.

We report here a case of neuropathic pain affecting the third branch of the trigeminal nerve, caused by periodontitis associated with a left low-level impacted mandibular third molar, accompanied by neuropathic pain in the second branch, potentially due to sensitization of the third branch. A rat study has shown that neuropathic pain in the third branch area of the trigeminal nerve can spread to the second branch region [6]. However, to our knowledge, no clinical cases demonstrating such complex symptomatology have been reported. We analyzed the clinical symptoms considering these possible underlying mechanisms.

## Case presentation

A 57-year-old woman with no relevant medical or family history visited a dental clinic complaining of persistent pain in the left buccal region. She was diagnosed with caries in the left maxillary second molar and underwent pulpectomy. Following the procedure, the pain in the left buccal region resolved. However, six months later, she developed persistent pain in the left maxillary second molar along with burning pain extending from the left buccal area to the mandible. She then visited another dental clinic, where she was diagnosed with left mandibular pericoronitis. Because pus drainage was observed from the distal periodontal pocket of the left mandibular second molar, she was prescribed antibiotics. One month later, the drainage ceased, and the pericoronitis of the left mandibular third molar improved, but the burning pain in the left mandible persisted. She also experienced dull pain in the left maxillary second molar that did not improve after re-root canal treatment.

She was subsequently referred to our hospital for further evaluation and management of her pain and was examined by an orofacial pain specialist. At the initial examination, she reported burning pain from the left buccal region to the mandible, dull pain in the left maxillary second molar, and persistent allodynia of the gingiva in the same area (Numerical Rating Scale: 6). Intraoral examination revealed percussion pain and allodynia of the gingiva surrounding the left maxillary second molar, which was undergoing root canal treatment. Additionally, pus drainage from the periodontal pocket of the left mandibular second molar was noted. No percussion pain or gingival swelling was detected in other teeth. Extraoral examination revealed tenderness and stiffness of both masseter muscles; however, this pain differed from her chief complaint and was not associated with referred pain. No swelling or tenderness was found in the submandibular lymph nodes, and no hypoesthesia was present in the mental region.

**Figure 1:**
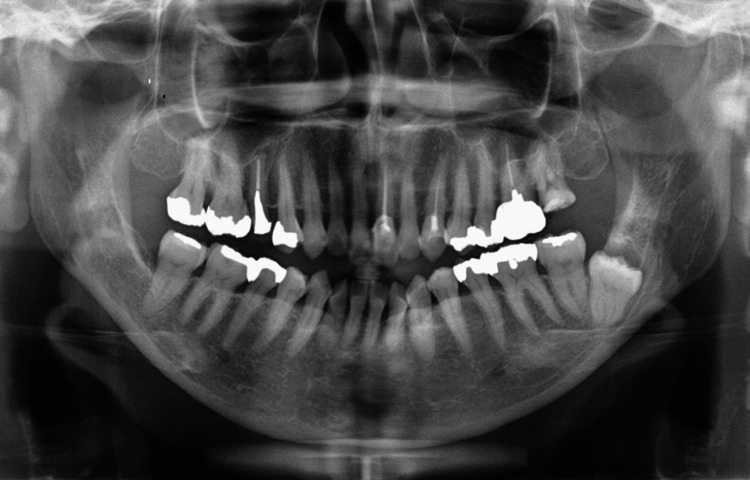
Panoramic radiograph at the initial examination The root of the left mandibular second molar is positioned very close to that of the mandibular third molar; however, no root resorption was observed in the left mandibular second molar. Additionally, no obvious apical lesions were detected in the left maxillary second molar.

Blood tests showed no evidence of systemic inflammation. A panoramic radiograph demonstrated that the left mandibular third molar was minimally impacted and in close proximity to the inferior alveolar nerve. The crown was surrounded by a periapical radiolucency (Figure 1).

Based on her clinical history and findings, we diagnosed the cause of her pain as pericoronitis of the left mandibular third molar, accompanied by neuropathic pain in the left third branch of the trigeminal nerve originating from inflammation due to pericoronitis. Although the pus from the periodontal pocket resolved, both symptoms persisted. To further clarify the diagnosis, local infiltration anesthesia with 2% lidocaine containing epinephrine (1:80,000) was administered around the apical region of the left maxillary second molar, and a mandibular nerve block with 2% lidocaine was performed in the left mandibular molar area. Following these procedures, the burning pain from the left buccal region to the mandible and gingival allodynia around the left maxillary second molar remained unchanged, but the dull pain in the same area disappeared. Based on these results, the persistent burning pain was considered to be left trigeminal neuropathic pain. Treatment with pregabalin was initiated, titrated to 100 mg/day, and maintained for one month. Subsequently, the pain resolved. The pregabalin dose was then gradually tapered and discontinued.

**Figure 2:**
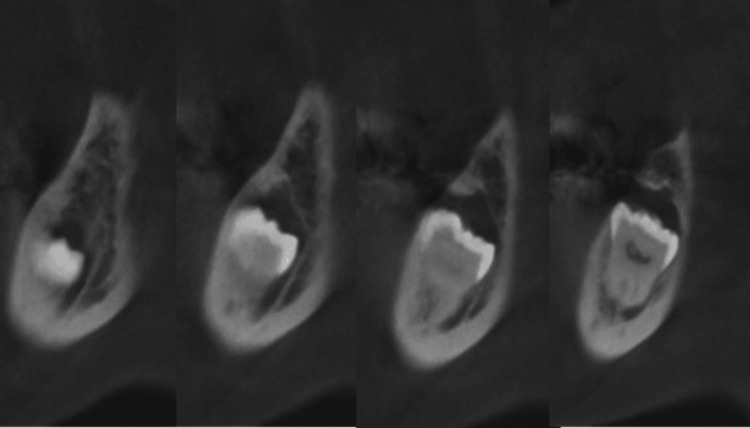
Sagittal section of the CT scan The left mandibular third molar is positioned superior and close to the mandibular canal. A periapical radiolucency is visible around the crown apex.

After her pain was relieved, oral surgeons were consulted to extract the left mandibular third molar, which was identified as the source of her pain. CT revealed that the left mandibular third molar was impacted at a low level and associated with a follicular dental cyst (Figure 2). The tooth was extracted under general anesthesia.

**Figure 3:**
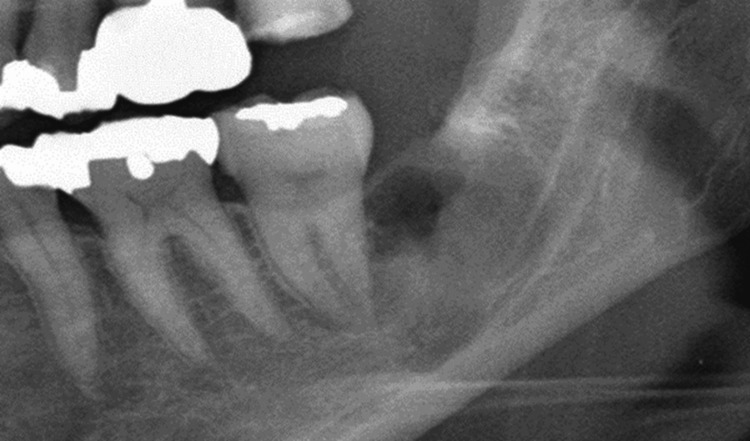
Panoramic radiograph taken one month after tooth extraction, showing a highly magnified view of the extraction site The panoramic X-ray taken one month after extraction showed an indistinct maxillary margin of the mandibular canal.

During the operation, although the mandibular canal was visible beneath the socket floor through thin bone, there was no obvious exposure of the inferior alveolar nerve or artery. One week after surgery, the postoperative hypoesthesia in the chin area and the postoperative pain both resolved. One month later, the wound had healed; however, the burning pain recurred from the left buccal region to the mandible. Simultaneously, the dull pain and gingival allodynia in the left maxillary second molar area also returned, reaching the same intensity as initially observed (Figure 3).

**Figure 4:**
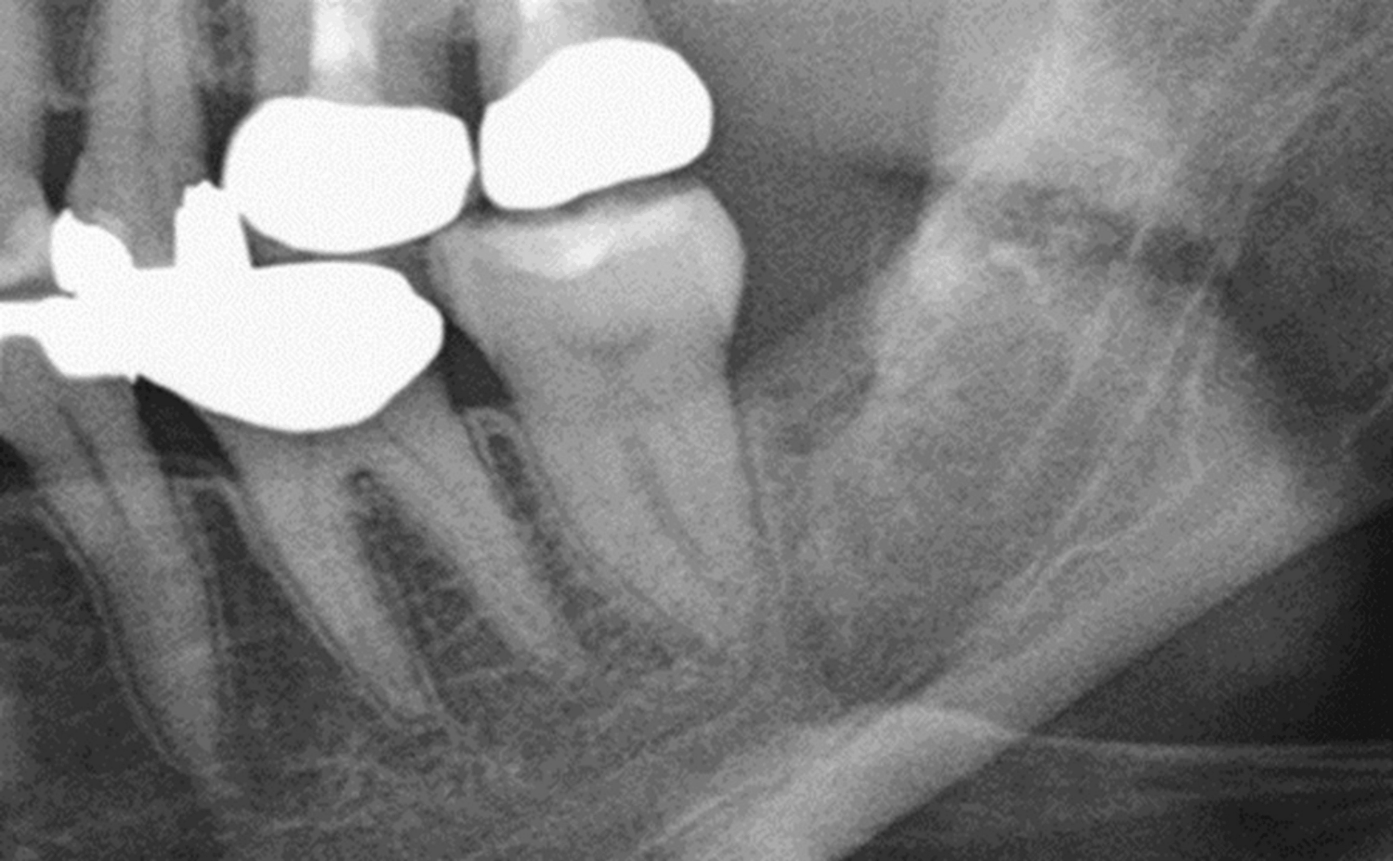
Extraction socket one year after tooth removal Bone regeneration was evident in the extraction socket one year after surgery. Additionally, the maxillary margin of the mandibular canal appeared clearer compared to immediately after extraction.

This phenomenon was diagnosed as neuropathic pain due to sensitization of trigeminal neurons, and pregabalin treatment was restarted. Pregabalin was titrated up to 100 mg/day but had no analgesic effect. Therefore, amitriptyline was added to the regimen. When the amitriptyline dose reached 80 mg/day, her burning pain was relieved, the dull pain in the left maxillary second molar resolved, and the root canals were successfully filled. One year later, healing of the extraction socket was confirmed (Figure 4).

Her burning pain from the left buccal region to the mandible persisted but was less severe than initially, allowing her to discontinue pregabalin. Amitriptyline was gradually tapered over six months and stopped once her pain had significantly decreased. By that time, the dull pain in the left maxillary second molar had resolved. Her pain remained well controlled, and follow-up was completed.

## Discussion

Neuropathic pain is classified according to the cause or anatomical distribution of the damage. Inflammation-derived neuropathic pain originating from acute local inflammation differs from traumatic neuropathic pain by presenting with severe initial pain and progressive sensory symptoms. It often manifests as spontaneous burning pain, hypersensitivity to touch, and aching attacks [7]. Distinguishing burning pain or allodynia caused by neuropathic pain from the acute pain or dull sensation of mandibular osteomyelitis in the molar region is crucial. Accurate diagnosis of neuropathic pain based on symptoms and early treatment can promote a favorable healing process [8].

In the present case, pericoronitis of the left mandibular third molar was initially suspected and treated with antibiotics to reduce inflammation. Although the pericoronitis improved, burning pain persisted from the left buccal region to the mandible, confirming that inflammation of the left mandibular third molar caused inflammation-derived neuropathic pain in the third branch of the trigeminal nerve. Peripheral neuropathic pain typically occurs as spontaneous pain or hyperalgesia induced by stimulation following injury or degeneration of sensory nerves. Neuronal function depends not only on the neurons themselves but also on supporting glial cells and their interacting environment. A previous study examining nerve biopsies after postoperative neuropathic pain found mononuclear inflammatory infiltrates in both epithelial and intimal vessels [8].

Moreover, in this case, the dull pain in the left maxillary second molar flared alongside burning pain from the left buccal region to the mandible. Notably, only the dull pain in the left maxillary second molar was relieved by diagnostic local anesthesia. This suggests that the pain was not due to local inflammation but rather hypersensitivity of neuropathic pain spreading from the third to the second branch of the trigeminal nerve [9]. Tseng et al. reported that damage to one trigeminal nerve branch in rats can sensitize other branches [10]. Both increased sensitivity in damaged neurons and sensitization of undamaged neurons contribute to persistent pain [11].

The underlying mechanism of trigeminal neuralgia or allodynia associated with orofacial inflammation likely involves neuroplastic changes within the trigeminal ganglion (TG), trigeminal spinal subnucleus caudalis (Vc), and upper cervical spinal cord (C1/C2) [11]. Trigeminal nerve injury activates and causes accumulation of non-neuronal cells, such as satellite cells and macrophages in the TG, as well as microglia, astrocytes, and oligodendrocytes in the Vc and C1/C2. These non-neuronal cells release molecules that hyperactivate nociceptive neurons in these regions. In turn, hyperactivated nociceptive neurons release molecules that further activate non-neuronal cells, creating a crosstalk loop that leads to the over-activation of nociceptive neurons in the TG, Vc, and C1-C2 [12,13].

Neuropathic pain following dental treatment such as mandibular third molar extraction has been reported in 0.38% to 5.0% of patients [1,2,14]. However, to our knowledge, there are no previous reports of pericoronitis causing inferior alveolar neuropathic pain accompanied by hypersensitivity in other branches of the trigeminal nerve. The present case is the first clinical report supporting this possibility. Although the complex pathophysiology made diagnosis challenging, early recognition and appropriate treatment were achieved.

Furthermore, the recurrence of burning pain in the left mandible and allodynia in the gingiva of the left maxillary second molar raised the possibility that stimulation of the third branch by the extraction procedure caused acute traumatic neuropathic pain, which in turn sensitized the second branch of the trigeminal nerve [15]. While residual nociceptive pain due to mechanical stimulation during root canal treatment, remaining pulp, perforation, or root fracture cannot be excluded, the clinical course suggested that the original inflammation-derived peripheral neuropathic pain was exacerbated by local trauma from tooth extraction, long-term pain memory, and emotional factors. These combined to produce a complex pathophysiology.

In other fields, post-mastectomy pain syndrome (PMPS) presents with prolonged postoperative pain and numbness in the breast area after breast cancer surgery. Neuropathic pain is the primary cause of PMPS, affecting approximately 25-60% of patients postoperatively. Multiple factors, such as postoperative physical changes due to chemotherapy, radiotherapy, endocrine therapy, and anxiety about recurrence, complicate the pathophysiology [15-17]. In the present case, involvement of the amygdala and hippocampus, key regions for memory and emotion, may have influenced pain prolongation and recurrence through negative emotional processing [18].

The NICE guidelines and the Neuropathic Pain Special Interest Group recommend pregabalin (gabapentinoids), amitriptyline (tricyclic antidepressant), and duloxetine (selective serotonin-norepinephrine reuptake inhibitor, SNRI) as first-line pharmacological treatments for neuropathic pain [19-21]. Neuropathic pain is a complex condition often with low responsiveness to medication. Pregabalin is preferred as a first-line agent due to its lack of drug interactions, linear pharmacokinetics, and predictable dose-dependent effects. Doses between 150 and 600 mg/day demonstrate a satisfactory dose-response relationship, with a number needed to treat (NNT) between 6.5 and 9.4. Amitriptyline, administered at 25 to 150 mg/day, has moderate evidence supporting its use, with an NNT of 3.0 to 4.4 [22]. Pharmacological therapy was effective in our case.

One shortcoming in this case was the lack of prophylactic measures against neuropathic pain development following tooth extraction. Venlafaxine, an SNRI, has been reported to significantly reduce the incidence and intensity of chronic postmastectomy pain when used prophylactically [15]. Since extraction was planned at a site previously affected by neuropathic pain, preventative neuropathic pain medication should have been considered before surgery to reduce recurrence risk. Additionally, quantitative sensory testing could have been employed as an objective assessment tool not only at the initial visit but also throughout follow-up.

## Conclusions

We encountered a case of neuropathic pain in the trigeminal nerve caused by inflammation of the mandibular third molar. The diagnosis was challenging because the pain appeared to extend to the second branch of the trigeminal nerve, likely due to hypersensitivity. Additionally, the patient exhibited a complex condition in which symptoms in the second branch worsened after the extraction of the third molar. The pathogenesis of neuropathic pain is multifaceted, with many mechanisms still not fully understood. Therefore, early pain relief is crucial. Pain symptoms that do not follow the usual anatomical distribution should be carefully evaluated, and prevention and treatment strategies should consider the possibility of hypersensitivity.
